# Mobile Apps and Quality of Life in Patients With Breast Cancer and Survivors: Systematic Literature Review

**DOI:** 10.2196/42852

**Published:** 2023-07-26

**Authors:** Saeunn Rut Saevarsdottir, Sigridur Lara Gudmundsdottir

**Affiliations:** 1 University of Iceland Department of Health Promotion, Sport & Leisure Studies Reykjavik Iceland; 2 Sidekick Health Kopavogur Iceland

**Keywords:** mobile health, mHealth, breast cancer, quality of life, review, systematic review, cancer treatment, mobile app, patient care, survivorship, digital health intervention, lifestyle intervention, mobile phone

## Abstract

**Background:**

Side effects of breast cancer treatment may persist long into survivorship, reducing quality of life (QOL) in patients with breast cancer and survivors. There is growing evidence for the use of digital health technologies, such as mobile apps, to support self-management, decrease symptom burden, and improve QOL in patients with cancer. However, an updated overview of the effects of mobile apps on QOL and well-being in patients with breast cancer and survivors is needed.

**Objective:**

The aim of this review was to provide an overview of breast cancer–specific, mobile app–driven lifestyle or behavioral interventions in patient care through to survivorship and their impact on QOL and mental well-being.

**Methods:**

A systematic search of PubMed, Scopus, and Web of Science was conducted to identify relevant studies. The inclusion criteria were limited to original studies involving a trial of a mobile app–driven lifestyle or behavioral intervention for patients with breast cancer or survivors and using QOL or well-being measures. The results of the studies that met the inclusion criterion were then synthesized in text and table format. The quality of the evidence was assessed with the Cochrane risk-of-bias tool.

**Results:**

A total of 17 studies with the number of participants ranging from 23 to 356 met the inclusion criterion. Of the 17 reviewed studies, 7 (41%) delivered an app-only intervention, and 10 (59%) combined an app with additional supporting materials, such as SMS text messaging, telecoaching, wearables, or printed materials. Among the 17 reviewed studies, 6 (35%) focused on aiding patients with breast cancer during the active treatment phase (excluding ongoing hormone therapy), whereas the remaining 11 (65%) focused on survivorship. The majority of the studies (14/17, 82%) observed some positive effects on QOL or well-being measures.

**Conclusions:**

The results of the review indicate that mobile apps are a promising avenue for improving QOL and well-being in breast cancer care. Positive effects were observed in patients undergoing active treatment in all reviewed studies, but effects were less clear after chemotherapy and in long-term survivors. Although lifestyle and behavioral digital interventions are still being developed, and further research should still be pursued, the available data suggest that current mobile health apps aid patients with breast cancer and survivors.

## Introduction

### Background

Breast cancer remains the leading cause of cancer-related deaths among women worldwide [1]. Even so, thanks to improvements in early detection and breast cancer treatment, most Western countries have seen a reduction in mortality rates [1,2]. As a result, many breast cancer survivors live multiple productive years after treatment completion [3]. However, the majority of cancer treatments severely affect quality of life (QOL), causing both physical and psychosocial side effects that may disrupt patients’ physical function and mental well-being [4]. These side effects may persist and follow into survivorship. Physical symptoms, including chronic pain, paresthesia, allodynia, menopausal symptoms, weight problems, and fatigue, along with psychological distress, including the fear of recurrence, sleep disturbances, sexual issues, and cognitive problems, often persist [5-7]. Furthermore, evidence suggests that breast cancer survivors are at an increased risk of adverse mental health outcomes such as anxiety and depression [8].

Various lifestyle and behavioral interventions have been shown to improve the side effects experienced during and after breast cancer treatment as well as improve survival outcomes [9-14]. Regular physical activity (PA) after diagnosis has been found to be inversely associated with both breast cancer mortality and all-cause mortality, and even low-volume PA has been found to improve survival outcomes compared with inactivity [15,16]. Furthermore, there is strong evidence supporting the positive effects of PA on cancer and treatment-related side effects [17,18]. Integrative therapies, such as yoga and mindfulness, have been found to have a positive effect, particularly on the psychological health of patients with breast cancer and survivors, improving mental health, sleep disturbances, and perceived stress [12,19-22]. Another important factor improving survival outcomes in patients with breast cancer is continued adherence to adjuvant endocrine therapy, yet nonadherence is common [23,24]. Behavioral interventions using reminders and encouraging self-reporting medication adherence have been shown to increase adherence to adjuvant endocrine therapy, thus improving survival outcomes [25]. However, with the increasing numbers of cancer diagnoses and cancer survivors, the resource needs cannot always be met, in particular in patients progressing from their primary to follow-up treatments. Thus, the development and validation of easily accessible treatments have become a crucial need for this patient group.

There is a growing body of evidence supporting the use of digital health technologies in cancer care, helping patients to self-manage symptoms and side effects, improving QOL, and potentially increasing survival outcomes [26,27]. Mobile health (mHealth), a subset of digital health, is defined as the use of mobile devices to support the delivery of health practices, often taking the form of a mobile app [28]. Compared with internet-based interventions, mobile apps are more user-friendly because they perform at a much faster speed than a website and may offer basic content and features even without an internet connection. Providers of mHealth interventions can easily send push notifications to users, and interaction-enabled features and gestures (tapping, dragging, holding, and swiping) are much more elaborative in an mHealth intervention than in an intervention accessed via an internet site where design and features are constrained by web browsers. A limitation of mHealth is the low regulatory approval [29]. Another subset of digital health, digital therapeutics (DTx), is however highly regulated, backed by clinical trials, and supported by real-world outcomes [30]. DTx apps deliver disease-specific interventions via high-quality software programs with the aim to treat, manage, or prevent disease through behavior changes [30-32]. With the majority of the world population being smartphone users, mHealth and DTx present a cost-effective solution to the growing need for accessible care [33,34], a solution considered by patients to be a useful complementary tool to usual care [35].

### Objectives

Prior reviews support the use of mHealth interventions in general cancer care, suggesting that they may support self-management as well as decrease symptom burden, fatigue, and psychological distress [36,37]. A recent meta-analysis concluded that the use of mHealth apps improves health-related QOL in patients with cancer, with the strongest evidence found for PA, cognitive behavioral therapy, and mindfulness [38]. However, the need for disease-focused solutions over generic ones has been emphasized [39]. In a 2017 review focusing on QOL in patients with breast and prostate cancer, only 5 studies met the inclusion criteria. The authors concluded that the majority of these studies were of low-to-medium quality and that there was a lack of rigorous trials [40]. A 2019 review summarizing the effects of available apps on various outcomes in breast cancer found indications of their effectiveness in promoting weight loss, improving QOL, and decreasing stress [41]. In a very recent publication, Patel et al [42] point out that digital innovation in oncology lags behind other therapeutic areas despite there being a high unmet need among patients, health care providers, and caregivers. Importantly, there is a lack of data on the impact of digital treatments on QOL, which challenges optimal treatment decision-making. Thus, with increasing numbers of breast cancer survivors, continual development and release of new breast cancer mHealth interventions, and the use of digital health apps being projected to rise rapidly, an updated synthesis of current research is timely. The aim of this review was to provide an overview of breast cancer–specific, mobile app–driven lifestyle or behavioral interventions in patient care through to survivorship and their impact on QOL and mental well-being.

## Methods

### Study Design

A systematic review of the literature was performed following the PRISMA-checklist (see Multimedia Appendix 1). The aim of a systematic review is to make available evidence more accessible by identifying, evaluating, and synthesizing the findings of relevant individual studies on a specific topic [43].

### Search Strategy

An extended bibliography search was conducted on March 21, 2022, on PubMed, Scopus, and Web of Science. The search focused on mHealth in patients with breast cancer and survivors and the effect on QOL. The following search query was used: ((“digital therapeutics”) OR (“digital health”) OR (mhealth) OR (“mobile application”) OR (app) OR (smartphone)) AND ((breast cancer) OR (breast neoplasm)) AND ((lifestyle) OR (“quality of life”) OR (QOL) OR (anxiety) OR (depression) OR (mental health)). The search was limited to include results that contained the word “trial”.

### Selection of Studies

No lower or upper published date limit was set on the search results. Only original research published in English was considered for inclusion. The inclusion criteria were further limited to research pertaining to mHealth, mobile apps, or DTx; breast cancer care or survivorship; and QOL or well-being. In this instance, lifestyle or behavioral interventions were defined as any intervention intended to modify or encourage certain behavior, such as exercise, nutrition, self-management, stress management, and medication adherence. Only studies using either direct QOL measurements or measurements related to mental well-being, such as anxiety or depression scales, were considered for inclusion.

Studies that did not include an intervention involving a mobile app and did not include patients with breast cancer or survivors as the study population were excluded. Only studies directly related to breast cancer care or survivorship were considered for inclusion. Studies involving mobile apps that were not directly applied to the study population (eg, health care professionals using mHealth apps to manage breast cancer care) were excluded, as were studies using an app without a lifestyle intervention (eg, symptom monitoring without self-management or lifestyle modification information). In addition, we excluded web-based interventions, telehealth interventions, and other digital health interventions that did not involve a mobile app. Furthermore, studies pertaining to app development were excluded. Only original research was included, and reviews, editorials, and protocols were excluded. Titles and abstracts were screened to identify appropriate articles. The initial screening and inclusion of articles was performed by SRS. Both authors independently reviewed the remaining articles for the final selection.

### Data Extraction

Data were extracted and inputted into a Google Sheets file (Google LLC) by 1 reviewer (SRS) and then verified by a second reviewer (SLG). The following data were extracted for each included study: author and year of study; location of study, study population, and number of participants; brief description of the intervention delivered, intervention duration, and, when applicable, the follow-up period; control condition (if any); measurements; and key outcomes. Any discrepancies were resolved by discussion.

### Quality Assessment of the Included Studies

The quality of each of the included studies was evaluated using the Cochrane Collaboration’s 6-domain tool for assessing risk of bias in randomized trials. Study characteristics are classified as having high, low, or unclear risk of bias [44]. For each study, all items were first rated by 1 reviewer (SRS) and compiled in a Google Sheets file. Any doubts over unclear items were resolved by discussion between the authors.

## Results

### Study Selection

A total of 346 articles were identified, of which, once duplicates were removed, 258 (74.6%) remained for primary analysis. Of these 258 articles, 55 (21.3%) were identified as involving a mobile app intervention with patients with breast cancer or survivors as the study population.

**Figure 1:**
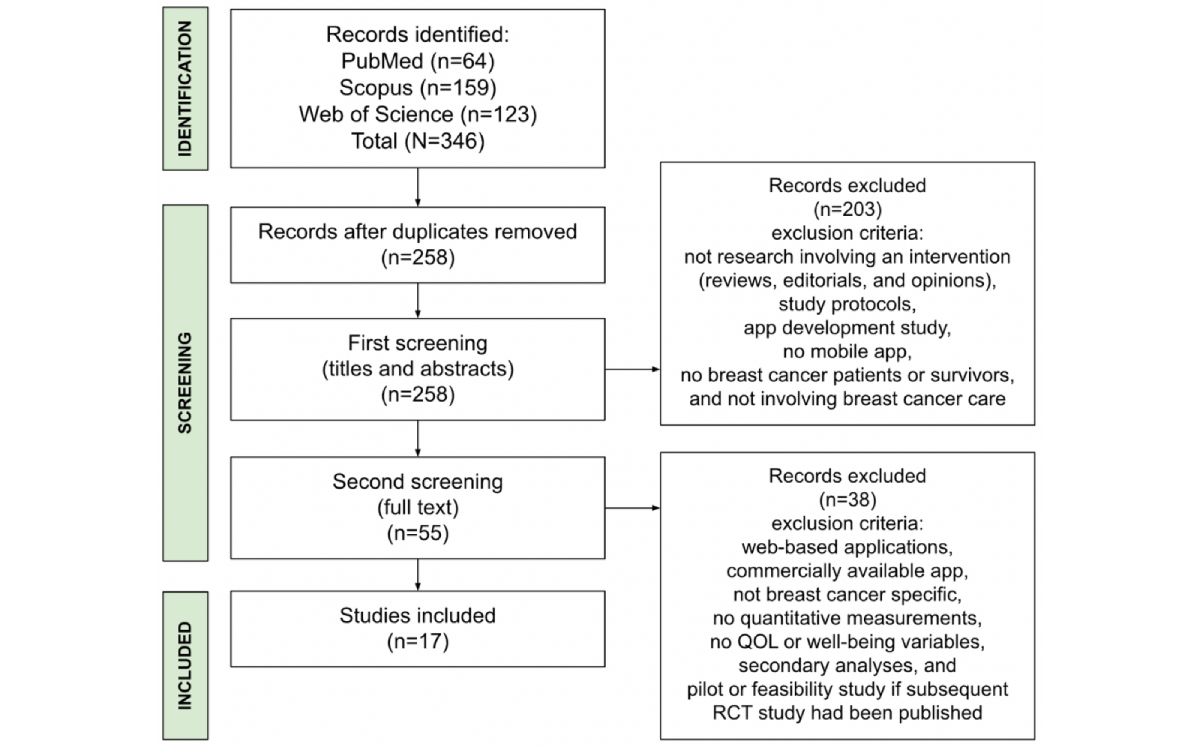
Flow diagram of the identification, screening, and inclusion of studies for review. QOL: quality of life; RCT: randomized controlled trial.

Subsequently, the full-text publications of these 55 articles were screened. In line with the defined exclusion criteria, the full-text screening excluded studies that were not breast cancer specific, involved commercially available health or wellness apps, were web based, had no quantitative measures, or had no QOL or well-being measures. In addition, we excluded interventions that involved smartphones or tablet devices but did not include a specific mobile app [45,46]. Some of the studies (2/55, 4%) were excluded because they did not have a lifestyle or behavior modification element despite using an app [47,48]. We also excluded studies where the feasibility of an app or the app functionality or engagement was being tested rather than the effectiveness of its contents on QOL and well-being [49,50]. Furthermore, all secondary analyses were excluded, as were pilot or feasibility studies if subsequent randomized controlled trial (RCT) studies had been published. Thus, of the initial 346 studies identified via database search, 17 (4.9%) were included in the final review. The flow diagram in Figure 1 details the study screening and selection process.

### Study Characteristics

The articles included were published between January 2017 and March 2022. Among the 17 articles, there were 10 (59%) RCTs, 5 (29%) pilot or feasibility studies, 1 (6%) nonrandomized controlled prospective cohort study, and 1 (6%) prospective quasi-randomized trial. All included articles studied an adult female population, either patients with breast cancer or survivors. The studies included were conducted in Asia (8/17, 47%), North America (5/17, 29%), and Europe (4/17, 24%). The sample size ranged from 23 to 356 participants, with 24% (4/17) of the studies having a sample of <50 participants, 35% (6/17) having a sample of between 50 and 100 participants, and 41% (7/17) having a sample of >100 participants. The majority of the studies (14/17, 82%) had a control group receiving care as usual or an active control group receiving a separate intervention.

Of the 17 reviewed studies, 7 (41%) delivered an app-only intervention, and 10 (59%) combined an app with additional supporting materials. The majority of the studies delivering a combined intervention used only 1 form of supporting material (7/10, 70%), whereas a minority used up to 3 forms of supporting materials (3/10, 30%). The supporting materials used included a combination of message-based reminders (SMS text message: 2/10, 20% and email: 1/10, 10%), wearables (pedometer: 1/10, 10% and Fitbit: 1/10, 10%), coaching calls (2/10, 20%), printed material (1/10, 10%), group support video calls (1/10, 10%), messaging group (1/10, 10%), other web-based material (2/10, 20%), and in-person rehabilitation sessions (1/10, 10%).

The reviewed articles all delivered interventions that had the goal, either primary or secondary, of improving QOL or mental well-being in some way, consistent with the inclusion criteria. The outcome measures varied across the studies, and the majority of the studies (15/17, 88%) measured more than 2 outcome variables. The most common measurements (6/17, 35%) used for QOL were the European Organization for Research and Treatment of Cancer Quality-of-Life Questionnaire Core 30 (EORTC QLQ-C30) and the European Organization for Research and Treatment of Cancer Breast Cancer–Specific Quality-of-Life Questionnaire (EORTC QLQ-BR23). Many of the studies (4/17, 24%) also used the Functional Assessment of Cancer Therapy-Breast (FACT-B) or the Functional Assessment of Cancer Therapy-Endocrine Symptoms (FACT-ES). For anxiety or depressive symptoms, the most common scales used (4/17, 24%) were the Hospital Anxiety and Depression Scale (HADS) and the State-Trait Anxiety Inventory (STAI). However, other measurements were also used. The length of the interventions varied from 3 weeks to 12 weeks, with a minority of the studies including a follow-up period after intervention completion (4/17, 24%).

All reviewed studies included a sample of women with breast cancer or breast cancer survivors. Of the 17 studies, 6 (35%) focused on aiding patients with breast cancer during the active treatment phase (excluding ongoing hormone therapy), whereas the remaining 11 (65%) focused on survivorship. Table 1 summarizes the study characteristics, intervention design, and study results.

**Table 1:** Description of study characteristics.

Study, year		Study design	Location, study population, and number of participants (n)	Intervention description, duration, and follow-up (when applicable)	Measurements	Control condition	Key outcomes
**Mobile apps during active treatment**							
	Ghanbari et al [51], 2021	RCT^a^	Iran; 82 patients with nonmetastatic breast cancer; intervention group: 41 and control group: 41	An mHealth^b^ self-management psychoeducational app for breast cancer care, including information on breast cancer and treatment, stress management techniques, self-esteem promotion, and anger management techniques; patients in the intervention group could interact with each other and with a psychiatric nurse; intervention duration: 4 weeks; no follow-up	Spielberger STAI^c^, RSES^d^, and an app satisfaction survey	Usual care	The intervention group showed a significant reduction in anxiety (*P*<.001) compared with the control group and a significant increase in self-esteem (*P*<.001) compared with the control group
	Fjell et al [52], 2020	RCT	Sweden; 149 patients with breast cancer undergoing neoadjuvant chemotherapy; intervention group: 74 and control group: 75	A cancer care mHealth app called Interaktor intended for early identification and management of symptoms as well as interaction with health care professionals; the app included self-reporting of symptoms, as well as education and information regarding symptom self-management; intervention duration: varied depending on length of chemotherapy; measurements were taken at baseline and 2 weeks after end of chemotherapy; no follow-up	MSAS^e^ and EORTC QLQ-C30^f^	Usual care	The intervention group reported significantly lower prevalence of symptoms on the QOL^g^ scales: nausea and vomiting (*P*=.007), appetite loss (*P*=.03), constipation (*P*=.007), and feeling sad, as well as significantly higher emotional functioning (*P*=.008) and lower symptom distress (*P*=.004)
	Hou et al [53], 2020	RCT	Taiwan; 112 newly diagnosed patients with breast cancer (stages 0-III); intervention group: 53 and control group: 59	An mHealth app intended for breast cancer self-management support, which included education, exercise and rehabilitation, diet and nutrition, emotional support, symptom and side effect tracking, social resources, and experience sharing, as well as expert consulting; intervention duration: 12 weeks, measurements at 6 and 12 weeks; no follow-up	EORTC QLQ-C30 and EORTC QLQ-BR23^h^	Usual care	At 12 weeks, both groups showed improvement in total QOL; the intervention group had a higher score in total QOL and total breast cancer–specific QOL; (*P*=.04) no difference was found at the 6-week measurement
	Grašič Kuhar et al [54], 2020	Nonrandomized controlled prospective cohort study	Slovenia; 104 patients with nonmetastatic breast cancer undergoing chemotherapy; intervention group: 55 and control group: 49	An mHealth app, mPro Mamma, that recorded symptoms and, depending on symptom severity, presented symptom self-management or advised seeking professional help, with symptom reports forwarded to an oncologist; intervention duration: varied depending on length of treatment, with measurements at 1 week after commencement of treatment and at completion of treatment; no follow-up	EORTC QLQ-C30, EORTC QLQ-BR23, and self-reported use of health care resources	Usual care	Global QOL (*P*=.02) and summary scores (*P*=.003) improved significantly in the intervention group compared with scores in the control group in the first week, and summary scores were still higher in the intervention group at the end of treatment (*P*=.002); no difference was observed in use of health care resources
	Zhu et al [55], 2018	RCT	China; 114 patients with breast cancer about to commence chemotherapy; intervention group: 57 and control group: 57	An mHealth breast cancer e-support app that included a learning forum, a discussion forum, an ask-the-expert forum, and a personal stories forum, as well as evidence-based information on breast cancer and symptom management; intervention duration: 12 weeks; 6-month follow-up	SICPA^i^, MSPSS^j^, MD Anderson Symptom Inventory, FACT-B^k^, HADS^l^, and app use data, including log-in frequency and use time	Usual care	Decrease in self-efficacy (*P*=.03) and QOL (*P*=.03) after chemotherapy was significantly less in the intervention group; symptom interference was significantly less in the intervention group (*P*=.02); no significant difference in symptom severity; no difference observed for social support, anxiety, and depression; at 6-month follow-up, no differences were found between the groups in any health outcomes
	Kim et al [56], 2018	RCT	South Korea; 76 patients with metastatic breast cancer about to receive chemotherapy; intervention group: 36 and control group: 40	A mobile game with the intention to improve symptom self-management and reduce side effects of chemotherapy, with educational content on side effects and their prevention, as well as management of an avatar that was rewarded for performing activities such as exercise; intervention length: 3 weeks; no follow-up	App satisfaction survey, use data, MARS^m^ (Korean version), BDI^n^, Spielberger STAI, and WHOQOL-BREF^o^ scale	Usual care	The intervention group showed increased QOL (*P*=.01), but no difference was found in depression or anxiety; in addition, the intervention group showed increased medication adherence compared with the control group (*P*<.001) as well as lower rates in reported adverse events (*P*=.02)
**Mobile apps for breast cancer survivors**							
	Ochi et al [57], 2022	RCT	Japan; 50 breast cancer survivors (stages I-IIa) who had recently completed primary treatment; intervention group: 25 and control group: 25	A home-based exercise program (*habit-B*), designed to incrementally increase level of strength, delivered via an mHealth app and a smartwatch, along with counseling or exercise guidance and support provided via email and the app; intervention duration: 12 weeks; no follow-up	Cardiorespiratory fitness (VO_2peak_^p^) measured by incremental multistage load on a bicycle ergometer, 6-minute walk test, 1-repetition maximum strength, chair-stand test, resting heart rate, blood pressure, body composition, Global Physical Activity Questionnaire score, Cancer Fatigue Scale-12, and health-related QOL; exercise adherence was measured via the app, and heart rate was measured using a smartwatch	Control group was given a smartwatch to wear but did not receive access to the app	No differences in QOL measurements or fatigue; the intervention group showed a significant improvement in VO_2peak_ and leg strength (*P=*.01), but no significant differences were found for other physical functions
	Fu et al [58], 2022	RCT	United States; 120 breast cancer survivors who had undergone surgical treatment at least 3 months before enrollment, reported pain, and may or may not report lymphedema symptoms or have a history of lymphedema; intervention group: 60 and control group: 60	A web- and mobile-based intervention called The-Optimal-Lymph-Flow (TOLF); the web-based platform included information on lymphedema, self-care, and lymphatic exercises, as well as a section where patients could ask an expert questions, whereas the mHealth app offered an additional way of practicing the lymphatic exercises; intervention duration: 12 weeks; no follow-up	Lymphedema and Breast Cancer Symptom Experience Index (Part I), limb volume difference measured with an infrared Perometer, and PIQ-6^q^, as well as height, body weight, BMI, and the Risk Reduction Behavior Checklist	Control group: access to web and mobile limb mobility exercises, with a focus on precautionary lifestyle behaviors	The intervention group reported significantly fewer cases and lower severity of chronic pain (*P*=.02), as well as fewer cases of arm or hand swelling (*P*=.04), heaviness (*P*=.03), redness (*P*=.03), and limited mobility in the shoulder (*P*=.02) and arm (*P*=.03); number of lymphedema symptoms did not differ between the groups
	Çınar et al [59], 2021	RCT	Turkey; 64 patients with breast cancer undergoing adjuvant endocrine hormone therapy; intervention group: 33 and control group: 31	An mHealth app with educational content, relaxation exercises, guided imagery, symptom diary, and question module where patients could send questions to specialist nurses; the app included RPM^r^; intervention duration: 12 weeks; no follow-up	FACT-ES^s^ quality-of-life scale, NCCN^t^ Distress Thermometer, and an app satisfaction survey	Usual care	The intervention group saw a positive impact on the majority of QOL measurements, including total (*P*=.003), emotional (*P*=.007), and functional well-being (*P*=.01), as well as endocrine symptom s (*P=*.004); no difference between the groups for social or family well-being scores; distress scores in the intervention group were lower than those in the control group (*P*=.004)
	Lozano-Lozano et al [60], 2020	RCT	Spain; 80 breast cancer survivors (stages I-IIIA) who were overweight or obese at time of diagnosis; intervention group: 40 and control group: 40	An mHealth app, BENECA, as well as a 2-month in-person group rehabilitation program; the app allowed for energy feedback monitoring through user-recorded daily dietary and physical activity habits; the rehabilitation program focused on symptoms, therapeutic exercises, and psychomotricity; intervention duration: 8 weeks; follow-up at 6 months	Primary measurements were EORTC QLQ-C30 (version 3.0) and EORTC QLQ-BR23; secondary measurements were DASH^u^, AROM^v^ of the shoulder using a goniometer, and upper body muscular strength using a digital handgrip; dual-energy x-ray absorptiometry was used to measure BMI, percentage fat mass, and bone mineral density	mHealth app and usual care	Both groups saw an improvement in QOL measures, but a more significant increase was seen in the intervention group (mHealth and rehabilitation); AROM and upper limb functionality were better in the intervention group (*P*<.001); effects were maintained at 6-month follow-up (*P*<.05), apart from those for emotional functioning, social functioning, systemic therapy side effects, and breast symptoms
	Yanez et al [61], 2020	Pilot study	United States; 80 Latinx breast cancer survivors (stages 0-III) within 2 to 24 months of completing primary treatment; intervention group: 40 and control group: 40	An mHealth app, MyGuide, designed to reduce symptom burden and improve health-related QOL through coping strategies, ongoing medication adherence, cancer knowledge, optimizing social support, psychosocial adaptation, and stress management; participants also received telecoaching; intervention duration: 6 weeks; follow-up at 2 weeks	BCPT^w^ symptom checklist (25 items), FACT-B (36 items), as well as acceptability and feasibility	An mHealth app, MyHealth, promoting healthy lifestyle behaviors; controls also received telecoaching	No difference was observed between the groups; improvements in breast cancer well-being were observed in both groups and maintained at the 2-week follow-up (*P*=.02); breast cancer symptom burden declined at the end of the intervention in both groups (*P*=.03) but was not sustained at the 2-week follow-up
	Nápoles et al [62], 2019	Feasibility study	United States; 23 Spanish-speaking breast cancer survivors who completed primary treatment within 1 year	A Spanish language mHealth app, trackC, with integrated activity tracker (Fitbit Zip), a hard copy survivor care planning program and information booklet, and health coaching telephone calls; the app provided information on potential side effects, healthy lifestyles, and survivorship resources; intervention duration: 2 months; no follow-up	Primary measurements: adapted version of the PROMIS^x^ Cancer Fatigue Scale, adapted version of the MOS^y^ Health Distress Scale, knowledge of cancer questionnaires, and an 8-item self-efficacy scale; secondary measurements: the 6-item Emotional Well-Being Scale from the Functional Assessment of Cancer Treatment-General, the Patient Health Questionnaire (8-item version), and the 6-item Brief Symptom Inventory Somatization Scale	N/A^z^	Fatigue (*P*=.02) and health distress (*P*=.01) levels were lower, self-reported knowledge of cancer care and resources increased (*P*=.03), and emotional well-being (*P*=.02) and daily steps (*P*=.02) increased; no change was found in the other secondary measurements or in self-efficacy
	Krok-Schoen et al [63], 2019	Pilot study	United States; 39 postmenopausal breast cancer survivors (stages 0-III) who had completed primary treatment and were eligible for receiving adjuvant hormone therapy	Daily medication reminders as well as weekly medication adherence reporting reminders via SMS text message; medication adherence was reported using an mHealth adherence app; intervention duration: 3 months; no follow-up	BCPT symptom checklist, Concerns About Recurrence Scale, 12-item Communication and Attitudinal Self-Efficacy-cancer scale, Center for Epidemiologic Studies Depression Scale, Brief Pain Inventory interference subscale, 7-item Fatigue Symptom Inventory interference subscale, Short Form-8 Health Survey (version 2), 14-item Perceived Stress Scale, 5-item Social Desirability Response Set, 20-item MOS Social Support Questionnaire, and medication adherence	N/A	No changes were observed for depressive symptoms, fatigue, pain interference, and physical functioning; however, study power was insufficient for these end points; improvements were observed in mental health functioning (*P*=.007) and perceived stress (*P*=.04); self-reported medication adherence increased (*P*=.02); and decreases in estradiol, estrogen, and estrone hormone levels were observed from baseline (*P*<.001; supporting the accuracy of the self-reported medication adherence scores); no other changes were observed; the majority of participants (*P*<.001) and physicians (100%) reported satisfaction with the intervention
	Imai et al [64], 2019	Pilot study	Japan; 38 breast cancer survivors >6 months after surgery	Problem-solving therapy delivered via an mHealth app aimed to reduce the fear of recurrence; intervention duration: 8 weeks; no follow-up	Concerns About Recurrence Scale (Japanese version), HADS, EQ-5D, Functional Assessment of Chronic Illness Therapy–Spiritual Well-being 12-item Scale, Supportive Care Needs Survey-Short Form 34, and Social Problem-Solving Inventory–Revised Short Form	N/A	Improvements were observed for the index score (*P*=.02) and the visual analog scale score (*P*=.002) on the EQ-5D; spiritual well-being scores (Functional Assessment of Chronic Illness Therapy–Spiritual Well-being 12-item Scale) increased (*P*=.01); psychological subscale (*P*=.05) and physical and daily living subscales (*P*=.003) scores of the Supportive Care Needs Survey-Short Form 34 reduced; and overall fear of recurrence decreased (*P*=.01); no other measurements observed reached significance
	Visser et al [65], 2018	RCT	The Netherlands; 122 survivors of nonmetastatic breast cancer who had completed primary treatment; intervention group: 63 and control group: 59	Face-to-face group medical consultation as well as access to mHealth app (myGMC); each patient received an iPad with the myGMC app, video chat app FaceTalk, reading app (iBooks), and contact information app (Contacts); the myGMC app included 13 short videos of interviews with breast cancer survivors; the other apps were used for online support group sessions, survivorship information, and contact information for the other members of the support group (a clinical nurse and the researcher); intervention duration: 3 months; follow-up at 6 months	The Symptom Checklist-90, the Empowerment Questionnaire for patients with breast cancer, the 8-item Cancer Worry Scale, EORTC QLQ-C30 (version 3.0) and EORTC QLQ-BR23), and MARS; in addition, feasibility, acceptability, and practicability of myGMC were measured, along with self-reported use statistics	Usual care	QOL did not differ between groups and neither did cancer worry, distress, or empowerment; improvements in medication adherence were observed but not sustained at the 6-month follow-up; more peer support was reported in the peer-support meetings than in the online support group meetings; overall app satisfaction was low
	Graetz et al [66], 2018	Randomized pilot study	United States; 48 breast cancer survivors (stages 0-III) who had completed primary treatment and been prescribed aromatase inhibitors; intervention group: 23 and control group: 25	An mHealth app along with weekly reminders via SMS text message, email, or both; the app encouraged tracking medication adherence and treatment-related symptoms; intervention duration: 8 weeks; no follow-up	Morisky Medication Adherence Scale-4, FACT-ES, app use log, and user feedback	App only	Both groups showed an increase in symptom burden after initiation of aromatase inhibitors; although the increase was less in the intervention group, it did not reach statistical significance; the intervention group had a higher proportion of app log-ins and higher medication adherence than the control group’ participants reported satisfaction with the app
	Uhm et al [67], 2017	Prospective quasi-randomized trial	South Korea; 356 breast cancer survivors who had completed primary treatment; intervention group: 179 and control group: 177	An mHealth app and pedometer with a 12-week regimen of aerobic and resistance exercise; intervention duration: 12 weeks; no follow-up	Various physical measurements, International Physical Activity Questionnaire–Short Form, EORTC QLQ-C30 and EORTC QLQ-BR23, and mHealth user satisfaction survey	Same 12-week regimen of aerobic and resistance exercise but in brochure form	Physical functioning, QOL, and self-reported physical activity increased in both groups; the intervention group reported high satisfaction regarding the mHealth app

^a^RCT: randomized controlled trial.

^b^mHealth: mobile health.

^c^STAI: State-Trait Anxiety Inventory.

^d^RSES: Rosenberg Self-Esteem Scale.

^e^MSAS: Memorial Symptom Assessment Scale.

^f^EORTC QLQ-C30: European Organization for Research and Treatment of Cancer Quality-of-Life Questionnaire Core 30.

^g^QOL: quality of life.

^h^EORTC QLQ-BR23: European Organization for Research and Treatment of Cancer Breast Cancer–Specific Quality-of-Life Questionnaire.

^i^SICPA: Stanford Inventory of Cancer Patient Adjustment.

^j^MSPSS: Multidimensional Scale of Perceived Social Support.

^k^FACT-B: Functional Assessment of Cancer Treatment-Breast.

^l^HADS: Hospital Anxiety and Depression Scale.

^m^MARS: Medication Adherence Rating Scale.

^n^BDI: Beck Depression Inventory.

^o^WHOQOL-BREF: World Health Organization Quality of Life-Brief Version scale.

^p^VO_2peak_: peak oxygen uptake.

^q^PIQ-6: Pain Impact Questionnaire-6.

^r^RPM: remote patient monitoring.

^s^FACT-ES: Functional Assessment of Cancer Therapy-Endocrine Symptoms.

^t^NCCN: National Comprehensive Cancer Network.

^u^DASH: Disabilities of the Arm, Shoulder, and Hand.

^v^AROM: active range of motion.

^w^BCPT: Breast Cancer Prevention Trial.

^x^PROMIS: Patient-Reported Outcomes Measurement Information System.

^y^MOS: Medical Outcomes Study.

^z^N/A: not applicable.

### Mobile Apps During Active Treatment

Symptom management was the primary focus for the majority of the articles identified (5/6, 83%) that delivered an intervention during active treatment [52-56]. However, many of the interventions (3/6, 50%) also connected patients with health care professionals [51-53], some (2/6, 33%) focused on mental health [51,55], some (3/6, 50%) focused on social support [51,53,55], and 1 (17%) of these 6 studies included a component on exercise and nutrition [53]. All studies used an mHealth app, one of which was a mobile game–based app [56]; however, none of the studies specified using a DTx app. All studies indicated some positive effects on QOL or well-being measurements [51-56].

Of the 6 studies, 4 (67%) specifically related to patients with breast cancer undergoing chemotherapy [52,54-56]. Fjell et al [52] randomized 149 patients with breast cancer undergoing neoadjuvant chemotherapy to receive access to an mHealth app—Interaktor—or care as usual. Patients self-reported symptoms within the app, which offered education on symptom self-management and remote patient monitoring (RPM). At the end of the intervention duration, which varied depending on each patient’s chemotherapy schedule, the intervention group reported significantly lower prevalence of chemotherapy-related side effects, higher emotional functioning, and lower overall symptom distress as well as physical symptom distress [52]. Zhu et al [55] randomized 114 patients with breast cancer about to commence chemotherapy to receive a breast cancer e-support mHealth app or care as usual for 12 weeks. The app was intended for education, symptom management, and social support. It included a learning forum, a discussion forum, a personal stories forum, and an ask-the-expert forum. After commencement of chemotherapy, a decrease in QOL and self-efficacy was observed in both groups, but the decrease was significantly less in the intervention group, as was the decrease in symptom interference. No significant difference was observed for symptom severity, social support, anxiety, or depression. At the end of the 6-month follow-up, no significant differences were observed between the groups [55]. Kim et al [56] randomized 76 patients with metastatic breast cancer to receive access to a mobile game app or care as usual for 3 weeks. The mobile game was intended to improve self-management and reduce chemotherapy-related side effects. The game included educational content as well as the control of a personalized avatar. There was an increase in medication adherence and QOL as well as lower rates in reported adverse events in the intervention group compared with the control group. No significant differences were observed for depression or anxiety. However, more than half of the participants in the intervention group reported finding the game difficult to use [56]. In their prospective controlled cohort study, Grašič Kuhar et al [54] gave the intervention group (n=55) access to an mHealth app, mPro Mamma. The app allowed for recording of symptoms and severity and delivered symptom self-management techniques and RPM. The control group had undergone treatment in the year prior, when the app was not yet fully developed. At the end of treatment, the QOL summary scores were significantly higher in the intervention group than in the control group [54].

Hou et al [53] focused on newly diagnosed patients with breast cancer, randomizing 112 participants to receive a symptom self-management app or care as usual for 12 weeks. The app included educational content, exercises, dietary advice, symptom tracking, emotional support, and experience sharing, as well as expert consulting. Although both groups reported an increase in total QOL EORTC QLQ-C30), the increase was significantly more in the intervention group, as was the increase in functional scores [53].

One of the studies delivering mHealth alongside clinical care focused primarily on mental health. Ghanbari et al [51] randomized 82 patients with breast cancer to receive either an intervention (a self-management psychoeducational app) or care as usual. The app included educational information as well as stress and anger management techniques, along with self-esteem promotion. In addition, there was a social support group on the messaging app WhatsApp, where the patients in the intervention group could interact with each other as well as with a psychiatric nurse. After the 4-week intervention, the intervention group showed a significant reduction in anxiety (state, trait, and total) as well as a significant increase in self-esteem when compared with the control group [51].

### Mobile Apps in Survivorship

The intervention focus varied for the reviewed studies focusing on mobile apps in survivorship. More than one-third (4/11, 36%) of the studies either focused on, or included an element of, PA as part of the intervention [57,60,62,67]. Some of the studies (3/11, 27%) included a focus on symptom burden [59,61,62], 9% (1/11) of the studies included RPM [59], 18% (2/11) addressed medication adherence [63,66], 9% (1/11) focused on lymphedema management [58], 9% (1/11) focused on social support [65], and 9% (1/11) specifically addressed the fear of recurrence [64]. All studies used an mHealth app, most of them (9/11, 82%) in conjunction with supporting materials [57,58,60-63,65-67]; however, none of the studies specified using a DTx app.

The studies that focused on PA had mixed results regarding QOL measurements. Ochi et al [57] randomized 50 breast cancer survivors to receive an mHealth app and a smartwatch or a smartwatch alone for 12 weeks. The app delivered a home-based exercise program, *habit-B*, with exercises designed to incrementally increase the patients’ level of strength. The intervention group showed significant improvements in some physical functions, including aerobic capacity (peak oxygen uptake) and leg strength, compared with the control group. However, no significant differences were observed for QOL measurements [57]. In the RCT conducted by Lozano-Lozano et al [60], which involved 80 breast cancer survivors who were overweight or obese at diagnosis, both groups received access to an mHealth app, BENECA, but the intervention group also received an in-person rehabilitation program. The app encouraged monitoring of daily dietary and PA habits to monitor energy feedback. The rehabilitation program included group sessions that focused on symptoms, therapeutic exercises, and psychomotricity, which refers to movements intended to enhance potential development or recovery and encompasses aspects of self-awareness [68,69]. At the end of the 8-week intervention, an improvement in QOL measures was found in both groups, but the increase was significantly greater in the intervention group, as was upper limb functionality and active range of motion of the shoulder [60]. Uhm et al [67] compared a 12-week exercise regimen delivered through an mHealth app along with a pedometer with the same program delivered in brochure form in a sample of 356 breast cancer survivors. Both groups were found to have a significant increase in QOL, physical function, and self-reported PA. The difference between the groups was not significant [67].

Nápoles et al [62] had a mixed focus on symptom burden, self-efficacy, and PA in their feasibility study. In this small study, 23 Spanish-speaking breast cancer survivors received access to an mHealth app, trackC, with an integrated activity tracker, hard-copy informational material, and telecoaching. The app included individual treatment history as well as information on potential side effects, healthy lifestyles, and survivorship resources. The integrated activity tracker was intended to promote PA through daily step goals. At the end of the 2-month intervention, significant improvements were observed in fatigue, health distress, and emotional well-being compared with baseline. Daily steps and self-reported knowledge of cancer care and resources increased significantly. However, no significant change was observed in self-efficacy [62]. Another study focusing on Spanish-speaking breast cancer survivors also included a focus on symptom burden. In their pilot study, Yanez et al [61] randomized 80 Latina breast cancer survivors to receive 1 of 2 mHealth apps. The intervention group received access to MyGuide, an mHealth app designed to reduce symptom burden and improve QOL through educational content, medication adherence, social support and psychosocial adaptation, stress management, and coping strategies. The control group received access to MyHealth, an mHealth app designed to promote healthy lifestyle behaviors such as nutrition and PA. Both groups also received telecoaching. At the end of the 6-week intervention, an improvement in symptom burden was observed in both groups; however, no significant differences were observed between the groups [61].

Krok-Schoen et al [63] focused on increasing adherence to adjuvant hormone therapy. In this pilot study, 39 postmenopausal breast cancer survivors received daily SMS text message reminders as well as access to an mHealth app intended for self-reporting medication adherence. At the end of the 3-month intervention, self-reported medication adherence increased significantly from baseline, and blood tests confirmed lower hormone levels, suggesting accuracy in the self-report measurements. Moreover, significant improvements were observed in mental health functioning and perceived stress [63]. In their pilot study, Graetz et al [66] randomized 44 breast cancer survivors who had been prescribed aromatase inhibitors (AI) to have access to an app that encouraged tracking medication adherence and treatment-related symptoms. The intervention group had access to the app and also received weekly SMS text message or email reminders to use the app whereas the control group received app only. At the end of the 8-week intervention, both groups showed an increase in symptom burden after AI initiation; although the increase was less in the intervention group, it did not reach a statistical significance. The intervention group reported higher AI adherence and higher weekly app use [66].

Fu et al [58] focused on lymphedema prevention and management. Breast cancer survivors (n=120) reporting pain or lymphedema symptoms were randomized to 1 of 2 web- and mobile-based programs. The intervention group received a program called The-Optimal-Lymph-Flow (TOLF), which included web-based educational information and lymphatic exercises as well as an mHealth app for performing the exercises on the go. The control group received an arm precaution program with web-based information on precautionary lifestyle behaviors as well as mobility exercises, also accessible via an mHealth app. At the end of the 12-week intervention, the TOLF group reported significantly fewer cases and less severity of chronic pain than the arm precaution group. Furthermore, the TOLF group reported significantly fewer cases of arm or hand swelling, heaviness, redness, and limited mobility in the arm and shoulder. However, neither the mean number of lymphedema symptoms nor the cases of general bodily pain differed between the groups. Significant improvements were observed in QOL measurements in both groups [58].

The study by Visser et al [65], which included 122 breast cancer survivors, focused on psychosocial support and the efficacy of group medical consultations (GMCs). Participants were randomized to receive face-to-face GMCs along with access to an mHealth app, myGMC, or care as usual. The intervention group received an iPad with the myGMC app, along with a video chat app (FaceTalk), a reading app with additional information (iBooks), and a contact information app. The app included videos of interviews with breast cancer survivors. Online group support sessions were held via FaceTalk. No significant differences were observed between the intervention group and the control group in terms of measurements of distress, empowerment, cancer worry, or QOL; however, a small yet significant improvement was found in medication adherence in the intervention group. The intervention group reported significantly more peer support in the face-to-face meetings than in the online group support meetings, and overall app satisfaction was low [65].

Only 2 (18%) of the 11 studies delivered an app-only intervention to breast cancer survivors. Çınar et al [59] randomized 64 breast cancer survivors undergoing hormone therapy to an mHealth app intervention or a control group for 12 weeks. The app featured educational content, relaxation techniques, and a symptom diary, as well as daily personalized reminders related to patient care.A positive impact was observed on the majority of the QOL measurements (FACT-ES) as well as a significant reduction in distress levels in the intervention group compared with the control group [59]. Imai et al [64], who also delivered an app-only intervention, addressed the fear of recurrence. In their pilot study, 38 breast cancer survivors received problem-solving therapy (PST) through an mHealth app. The app included sessions outlining PST and its principles and skills. After gaining an understanding of PST, the participants used a worksheet within the app to work on problems that were triggering a fear of recurrence. At the end of the 8-week intervention, overall fear of recurrence reduced significantly from baseline [64].

### Risk-of-Bias Assessment

**Figure 2:**
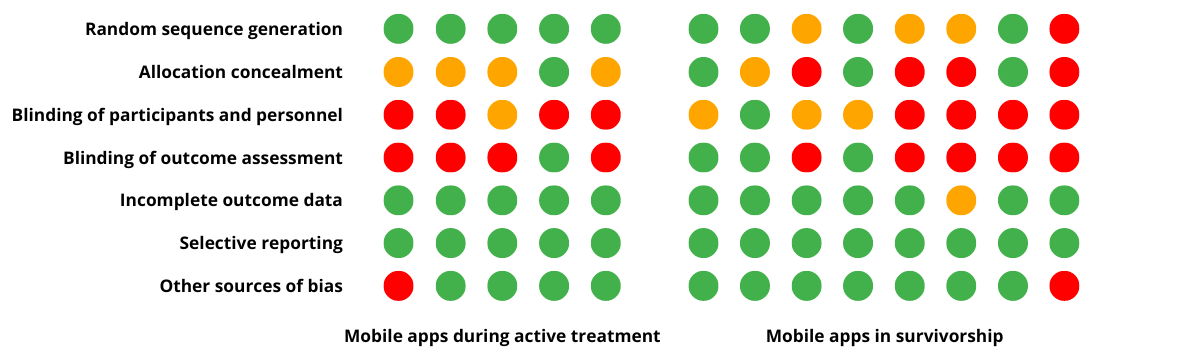
Assessment of risk of bias using the Cochrane Collaboration’s tool [51-53,55-61,65-67]. Green: low risk of bias, red: high risk of bias, and orange: unclear risk of bias.

Studies that included a randomization element were scored using the Cochrane risk-of-bias tool. The studies not included in the bias assessment were nonrandomized pilot and feasibility studies [62-64] and a nonrandomized prospective cohort study [54]. In all studies (n=13), at least 1 domain was scored as *high risk of bias* or *unclear risk of bias*. Among the 13 studies, random sequence generation was scored as high risk of bias in 1 (8%) and unclear risk of bias in 4 (31%). Allocation concealment was scored inadequate or unclear risk of bias for the majority of the studies (9/13, 69%). Blinding of participants and personnel was not performed or unclear in 11 (85%) of the 13 assessed studies. None of the studies were classified as high risk of bias for incomplete outcome data; however, 1 (8%) of the 13 studies was deemed to have an unclear risk of bias. No studies were deemed to have a high risk of bias for selective reporting. Of the 13 studies, 2 (15%) were considered to have a high risk of bias for other sources of bias. Risk of bias in multiple domains was present in the majority of the studies (8/13, 62%). The majority of the studies (10/13, 77%) were deemed to have an unclear risk of bias on at least 1 domain. The results of the risk-of-bias assessment are presented in Figure 2 [51-53,55-61,65-67].

## Discussion

### Principal Findings

This study synthesizes the literature on the effects of lifestyle interventions via mHealth apps on QOL and well-being in patients with breast cancer. As digital interventions and health apps continue to grow in popularity, critical reviews of the available evidence in this field must follow with the aim of easily accessible, evidence-based, and clinically accepted DTx becoming available to all patients with breast cancer and survivors. A total of 17 studies were identified that delivered a lifestyle or behavioral intervention to patients with breast cancer or survivors with the support of a mobile app, including measurements of QOL or well-being. All apps identified in the results were mHealth apps; however, none of the studies mentioned DTx, indicating that the interventions have not been through a regulated approval process. Overall, the results of the review indicate that mobile apps are a promising avenue for improving QOL and well-being in patients with breast cancer and survivors.

All mobile app interventions delivered during active treatment resulted in some form of positive effect on QOL or well-being. The majority of the studies (5/6, 83%) focused on symptom management [52-56]. Although some of the studies (2/6, 33%) included measurements for anxiety [55,56], only 1 (17%) of the 6 studies focused on mental health as the primary outcome [51] and was the only study to observe a significant reduction in anxiety. This may speak to a need for including mental health–focused elements in addition to symptom management when designing mHealth apps for patients with breast cancer.

Among the studies focusing on symptom management in patients with breast cancer, the majority (4/5, 80%) focused on patients undergoing chemotherapy [52,54-56]. However, only 1 (25%) of these 4 studies specifically addressed women with metastatic breast cancer [56]. This may point to a lack of available mobile apps that aim to improve QOL in women with advanced stage breast cancer.

Although all studies delivering a mobile app intervention during active clinical care observed positive effects on QOL or well-being, only 1 (17%) of these 6 studies included a long-term follow-up period [55]. At 6 months after active treatment, no significant differences were observed between the intervention and control groups. Although self-efficacy improved and QOL reduction was less in the intervention group, the positive effects were not sustained after completion of chemotherapy [55]. This may hint at either a need for continued support or an alternative intervention strategy after completion of chemotherapy. Furthermore, not all interventions covered the full duration of active clinical treatment, which, combined with the lack of follow-up, means that the long-term effects of the mobile app interventions cannot be accurately predicted. Similarly, for breast cancer survivors, only 2 (18%) of the 11 studies included a follow-up period [61,65]; of these 2 studies, 1 (50%) observed some sustained positive effect at the 2-week follow-up [61]. This highlights the need to gain further knowledge of the long-term effects of mobile app–driven lifestyle interventions on QOL and well-being in patients with breast cancer and survivors.

Overall, the results for breast cancer survivors varied more so than those for patients with breast cancer. Of the 3 studies focusing primarily on PA [57,60,67], only 2 (67%) observed a significant positive effect on QOL [60,67]; of these 2 studies, 1 (50%) also included an in-person rehabilitation program, to which some of the positive effects could be attributed [60]. Improvements in QOL were also observed when the intervention combined multiple factors affecting QOL, such as PA, reduction of symptom burden, and nutrition [61,62]. This might highlight a need for a multifaceted solution combining PA and symptom management strategies to improve QOL and well-being in breast cancer survivors.

Of the 6 studies involving patients with breast cancer, 5 (83%) delivered an app-only intervention [52-56], whereas of the 11 studies identified as aiding breast cancer survivors, only 2 (18%) delivered an app-only intervention [59,64]. The remaining studies used various supporting materials in addition to the app, such as wearables [57,62], printed materials [62], non–app-based reminders [57,63,66], web-based information [58,65], non–app-based coaching or group support [51,61,62,65], and an in-person group rehabilitation program [60]. Ease of use has been identified as an important preference when it comes to mHealth interventions in breast cancer care [70]. Using multiple supporting materials, such as printed materials, a website, and an app, could cause participants to feel overwhelmed or confused as to where to seek information and support. Considering that undergoing and recovering from breast cancer treatment can cause psychological distress [7,8], it is important to avoid potentially adding to this distress with overly complicated intervention materials. In addition, spreading out the information across multiple sources may lead to confusion, reduced motivation, and ultimately reduced long-term adherence. Furthermore, the majority of the studies (8/10, 80%) observed positive effects on QOL or well-being variables [58-64,67]; however, because of the various supporting materials used, the positive effects observed cannot be attributed to the mHealth apps alone. Equally, it may be worth researching which supporting materials could be incorporated into the app to aid ease of use. An important aspect is also the design of the mHealth app itself. A successful mobile intervention for breast cancer is dependent upon collaboration among the designers, patients, and various health professionals, including nurses [71].

In the studies focusing on patients with breast cancer, all control groups received the usual standard of care. However, for breast cancer survivors, the condition for the control group, when there was one, differed greatly. Many of the control groups also received access to an mHealth app, either an alternative app (2/8, 25%) [58,61] or the same app but without certain supporting materials (2/8, 25%) [60,66], and many observed an increase in QOL measures for both groups (3/8, 38%) [58,60,61]. However, this does warrant more research into identifying which aspects of the mobile app–driven interventions are the most effective in improving QOL and well-being as well as the ideal content for a placebo app.

Intervention length varied, ranging from 3 to 12 weeks for patients with breast cancer and 6 to 12 weeks for breast cancer survivors. All studies involving patients with breast cancer observed some positive effects on QOL or mental well-being, regardless of intervention length. In terms of breast cancer survivors, most of the studies (8/11, 73%) observed some positive effects on QOL or mental well-being. The shortest intervention [61] observed QOL improvements that were similar to those of the longer ones [58,60,62-64,67]. Further research is needed to determine the optimal intervention length during active treatment as well as the ideal length and focus of a long-term support program.

This is the first review to focus on breast cancer–specific mobile apps and their effect on QOL. The review is based on a strict search strategy and a clear focus on lifestyle interventions and QOL. However, certain limitations need to be addressed. Only English-language articles were included; we neither performed a statistical analysis nor evaluated the studies for quality. Furthermore, although the majority of the included studies (10/17, 59%) were RCTs, a number of them (5/17, 29%) were feasibility or pilot studies [61-64,66], which limits the strength of the results. The studies that included a randomization element were assessed for risk of bias, and the assessment revealed that the most common risk of bias identified was related to blinding. Blinding participants and researchers to allocation to study group is challenging in the case of a lifestyle intervention, in particular because knowledge of the optimum *dose* of such interventions is not known. Thus, designing a placebo version of a mobile app may not be feasible, and blinding may therefore be difficult. Despite the limitations of this review, the results indicate that the use of mobile apps to improve QOL and well-being in patients with breast cancer and survivors is promising. Further research that includes mental health and psychosocial components is needed. In addition, further research delivering mobile app interventions without supporting materials is needed to fully determine the effectiveness of such apps. Future research would benefit from investigating long-term effects of mobile app–delivered lifestyle interventions in both patients with breast cancer and survivors.

### Conclusions

On the basis of this new and updated synthesis of current research on mobile apps for QOL and well-being in breast cancer care, suggestive conclusions can be drawn despite limitations owing to methodological differences of the studies. In particular, positive effects on QOL were observed in patients undergoing active treatment in all reviewed studies, whereas the effects were less clear in the months after chemotherapy and in long-term survivors. This may indicate a need for better understanding of patient needs when developing mHealth interventions for long-term survivors in terms of personalized approaches and adaptive therapies that can adjust to the changing needs of cancer survivors. Lifestyle and behavioral digital interventions are still being developed, and future solutions may become more sophisticated; however, the available data suggest that current mHealth apps aid patients with breast cancer and survivors and that the likelihood of adverse effects is low. Therefore, although research and improvements should still be pursued, the use of mHealth solutions in breast cancer care should be encouraged because they provide a cost-effective and accessible way to improve QOL and well-being.

## Appendix

Multimedia Appendix 1 PRISMA (Preferred Reporting Items for Systematic Reviews and Meta-Analyses) checklist.

## Abbreviations

AI: aromatase inhibitors
DTx: digital therapeutics
GMC: group medical consultation
mHealth: mobile health
PA: physical activity
PST: problem-solving therapy
QOL: quality of life
RCT: randomized controlled trial
RPM: remote patient monitoring
TOLF: The-Optimal-Lymph-Flow

## Data Availability

The protocol and other material can be provided upon reasonable request.
